# The Effect of Weight Loss on Pediatric Nonalcoholic Fatty Liver Disease

**DOI:** 10.1155/2013/398297

**Published:** 2013-05-27

**Authors:** David E. St-Jules, Corilee A. Watters, Ken Nagamori, Jeremy King

**Affiliations:** ^1^Department of Human Nutrition, Food and Animal Science, University of Hawai'i at Manoa, 1955 East West Road, Agriculture Science 314 J, Honolulu, HI 96822, USA; ^2^Department of Pediatrics, Kapi'olani Medical Center for Women and Children, 1319 Punahou Street, Honolulu, HI 96826, USA

## Abstract

This study evaluated the effect of weight loss on pediatric nonalcoholic fatty liver disease (NAFLD). Subjects included 81 overweight NAFLD patients referred to two pediatric gastroenterologists from 2000 to 2010. Data on subjects were obtained from review of medical charts. The effect of weight loss was assessed at 1–4 months, 5–8 months, 9–12 months, and beyond one year as the change in weight, BMI *z*-score (for age-and-sex), and alanine aminotransferase and the relationship between the change in body weight and BMI *z*-score, and the change in alanine aminotransferase. Subjects were mostly obese (99%), male (86%), and Asian (63%) and had median age of 14.1 (11.2–16.2) years and alanine aminotransferase of 105 (78–153) U/L at referral. Alanine aminotransferase decreased 32 ± 66 (*P* = 0.016), 30 ± 65 (*P* = 0.134), 37 ± 75 (*P* = 0.0157), and 45 ± 69 (*P* = 0.014) for subjects with follow-up data at 1–4 months (*n* = 47), 5–8 months (*n* = 26), 9–12 months (*n* = 19), and beyond one year (*n* = 19), respectively. During these time periods, neither was body weight (−0.2 to +7.1 kg) or BMI *z*-score (−0.12 to −0.05) significantly reduced, nor were changes in these variables associated with the change in alanine aminotransferase. These findings suggest that weight and BMI *z*-score may not be sufficient indicators of treatment response in pediatric NAFLD patients.

## 1. Introduction

Nonalcoholic fatty liver disease (NAFLD) is a common complication of pediatric obesity characterized by the inappropriate accumulation of fat in hepatocytes in the absence of other known causes of steatosis [1–3]. Hepatic steatosis is closely related to metabolic syndrome and may contribute to the pathogenesis of other obesity-related conditions [4–7]. Prior investigation of children diagnosed with metabolic syndrome in Hawai'i found that approximately two-thirds had elevated serum alanine aminotransferase (ALT) values suggestive of NAFLD [8]. 

The majority of children with NAFLD suffer from psychological, physical, and pain-related symptoms, which contribute to a lower physical and psychosocial health, and reduced quality of life [9]. When compared to obese controls, pediatric NAFLD patients were found to have greater depression and influence of body weight on self-esteem [10]. Moreover, hepatic steatosis may be accompanied by inflammation and/or fibrosis, which can progress to liver cirrhosis requiring transplantation [1, 11–13]. Along with this, children with NAFLD were found to have reduced age- and sex-standardized survival free of liver transplantation [13].

Similar to other obesity-related conditions, weight loss is the primary treatment strategy for overweight children with NAFLD and has been found to have good efficacy in clinical trials [14–16]. However, the effectiveness of weight reduction efforts on pediatric NAFLD in free-living individuals is not well established. One retrospective chart audit noted that nearly half (49%) of the pediatric NAFLD patients were able to lose 10% of their initial body weight, and that the vast majority of them (86%) saw a reduction in liver aminotransferases [13]. However, by the time of the final followup, most of these patients (76%) re-gained the lost weight with many patients (46%) having recrudescence of liver aminotransferases [13]. Weight loss treatment is particularly challenging to implement and evaluate in pediatrics because children are still developing, and changes in body weight may be confounded by increases in height. For this reason, age- and sex-standardized BMI should be used to monitor lifestyle interventions, especially in the long-term [15, 16].

The purpose of this study is to evaluate the effectiveness of weight loss on reducing serum alanine aminotransferase (ALT) in pediatric NAFLD. In addition to providing preliminary data on pediatric NAFLD in Hawai'i, this study will examine the relationship between changes in body weight and BMI *z*-score for age and gender, and serum ALT in the outpatient setting.

## 2. Materials and Methods

The source population for this study was patients referred to two pediatric gastroenterologists at Kapi'olani Medical Center for Women and Children (KMCWC) with elevated serum ALT or fatty liver between January 2000 and December 2010 (*n* = 124). Subjects with positive testing for hepatic viral infections, autoimmune hepatitis or metabolic liver diseases, or taking hepatotoxic medications (33/124 (27%)) were excluded. Additionally, as the primary purpose of this study was to evaluate the effectiveness of weight reduction on serum ALTs, subjects were excluded if they were not overweight (BMI < 85th percentile for age-and-sex; 2/124 (2%)), or had normal serum ALTs (<25.8 U/L for boys, <22.1 U/L for girls; 4/124 (3%)). The upper limit of normal for serum ALT was based on the 95th percentile for healthy boys and girls in the United States, which has been demonstrated to have superior sensitivity with only minor losses in specificity for diagnosis of NAFLD compared to the higher cutoffs used in most children's hospitals [21]. Finally, four subjects (3%) were missing data, leaving 81/124 (65%) of the source population available for analysis. The University of Hawai'i Committee on Human Studies and Hawaii Pacific Health Research Institute provided ethical approval for this study.

Demographic, anthropometric, biochemical, and clinical data on subjects were collected from medical charts. Body weight and height were used to calculate BMI *z*-scores according to the 2000 CDC growth charts for age and sex using the lambda, mu, and sigma (LMS) technique [17]. Systolic and diastolic blood pressures were converted into *z*-scores for age, sex, and height as per the National High Blood Pressure Education Working Group on High Blood Pressure in Children and Adolescents recommendations [18]. Self-reported ethnicity was available in the charts of 30/81 (37%) subjects. The remaining subject ethnicities were determined using the surname list method designed for the Multiethnic Cohort Study [22]. When a surname corresponded to more than one ethnic group, the subject was assigned to the dominant ethnicity in Hawai'i [23]. There were 11/40 (28%) subjects whose surnames were not found on the surname list. These subjects were considered White because both White and African American ethnicities are not represented in the surname list, and Whites are more common in Hawai'i [22, 23]. Subjects were then classified as American Indian, Asian (Chinese, Japanese, Korean, Filipino), Hispanic, Pacific Islander (Native Hawaiian, Samoan), or White as per the National Institutes of Health (NIH) guidelines for race and ethnicity [24]. 

**Figure 1:**
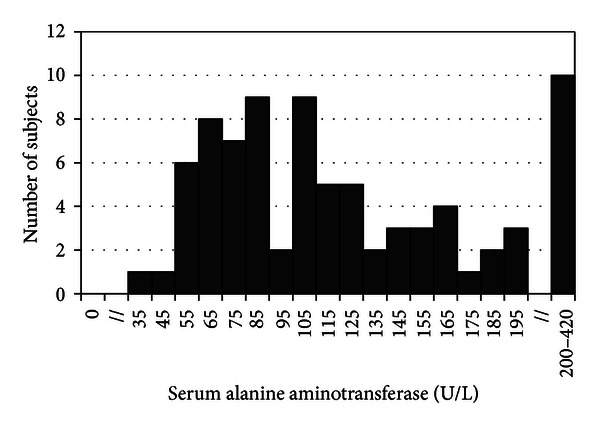
Frequency distribution of serum alanine aminotransferase (U/L) in subjects.

Characteristics of subjects at the time of referral to the gastroenterologist were summarized as frequency and percentages for categorical variables, mean and standard deviations for parametric continuous variables, and median and interquartile ranges for nonparametric continuous variables (Table 1). The designation of continuous variables as parametric (body weight, height, BMI *z*-score, HDL-cholesterol, and diastolic blood pressure) or nonparametric (age, fasting blood glucose, total- and LDL-cholesterol, triglycerides, systolic blood pressure, and alanine aminotransferase) was based on Shapiro-Wilk test and confirmed by analysis of frequency distribution graphs. The prevalence of comorbidities including obesity, fasting hyperglycemia, dyslipidemias, and hypertension was determined using previously published criteria in pediatric NAFLD patients (Table 1) [19, 20]. Serum alanine aminotransferase values of the sample at the time of referral to the gastroenterologist were presented in a histogram for visualization of frequency distribution (Figure 1). Liver biopsy was performed on a subset of subjects (*n* = 13), and the presence of inflammation and fibrosis was assessed based on liver histology reports. 

The effectiveness of weight loss intervention was assessed at time intervals of 1–4 months, 5–8 months, 9–12 months, and beyond one year as body weight, BMI *z*-score, and serum ALT using the independent two-sample *t*-test and Wilcoxon rank sum test for parametric and nonparametric continuous variables, respectively (Table 2). Only subjects with both anthropometric and serum ALT measurements obtained as part of the same clinic visit were included in this analysis. When a subject had more than one complete set of data for a given time interval, the visit furthest from the time of the initial consultation was used. The average change in subject height was also included to provide perspective on vertical growth. Sensitivity analysis was performed by comparison of subjects with follow-up data to those without follow-up data at each time interval using the independent two-sample *t*-test and Wilcoxon rank sum test for parametric and nonparametric variables, respectively (Supplementary Table 1 available online at http://dx.doi.org/10.1155/2013/398297). 

**Figure 2:**
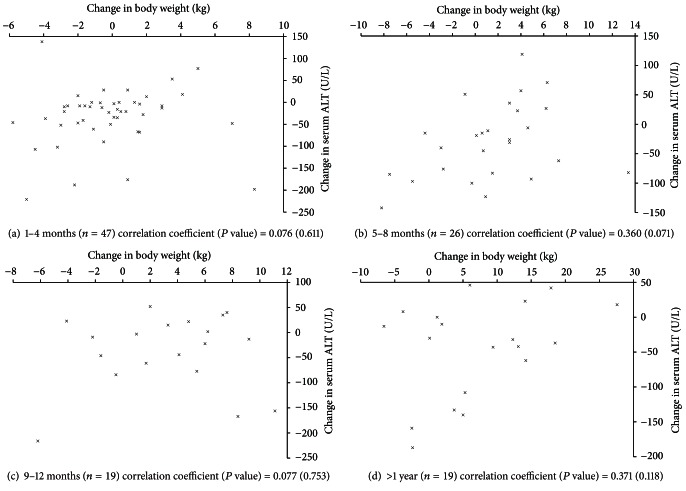
Scatterplot of subject's change in body weight and change in serum alanine aminotransferase at time intervals of 1–4 months, 5–8 months, 9–12 months, and greater than 1 year. ALT: alanine aminotransferase. The association between changes in body weight and changes in serum alanine aminotransferase was assessed by Pearson correlation, and is presented as correlation coefficient (*P* value).

The relationships between the changes in body weight and BMI *z*-score, and the changes serum ALT were examined at each time point using Pearson correlation and linear regression adjusting for initial serum ALT, and baseline values for body weight, and BMI *z*-score for the corresponding parameter (e.g., analysis of change in body weight and change in serum ALT included initial body weight and initial serum ALT as covariates) (Table 2). The changes in body weight and BMI *z*-score, and changes in serum ALT at each time interval were graphed as a scatterplot for visual presentation of data (Figures 1 and 2). Statistical tests were carried out using SAS v. 9.2, and graphs were created using Microsoft Excel for Mac 2011 v. 14.2.5. 

## 3. Results

The characteristics of the subjects at the time of referral are presented in Table 1. The majority of subjects were male (70/81 (86%)) and Asian (51/81 (63%)), and the median age was 14.1 years (interquartile range 11.2–16.2 years). Almost everyone had a BMI at or above the 95th percentile for age and sex (80/81 (99%)), and many had obesity-related comorbidities, although this could only be assessed in 27–34/81 (33–42%) and 59/81 (73%) of the subjects for laboratory and clinical parameters, respectively. Among the comorbidities assessed, hypertriglyceridemia (20/32 (63%)), hypercholesterolemia (21/34 (62%)), and low HDL-cholesterol (14/27 (52%)) were present in more than half of the subject tested, whereas hypertension (22/59 (37%)), high LDL-cholesterol (8/30 (27%)), and fasting hyperglycemia (6/33 (18%)) were less common. The serum ALT value of most patients was well above the upper limits of normal (Figure 1), and the median serum ALT was 105 U/L (interquartile range 78–153 U/L). Of the 13 subjects with liver biopsies, 13/13 (100%) had features of inflammation and 10/13 (77%) had fibrosis, which was noted to be at the stage of bridging fibrosis in three subjects (data not shown). 

Follow-up data was available for 47/81 (58%) subjects from 1–4 months, 26/81 (32%) subjects from 5–8 months, and 19/81 (23%) subjects from 9–12 months and beyond one year (Table 2). Baseline serum ALT was consistently higher in subjects that had follow-up data compared to those that did not, and this difference was statistically significant for the 1–4 months (*P* = 0.026) and 5–8 months (*P* = 0.026) time intervals and trended towards significance in subjects with follow up data beyond one year (*P* = 0.056) (Supplementary Table 1). In the 5–8 month time interval, baseline body weight (*P* = 0.024) and BMI *z*-score (*P* = 0.072) tended to be lower in the group that had follow-up data available (Supplementary Table 1).

Subject's gained weight consistently over the time intervals, but the BMI *z*-scores tended to be lower as this was at least matched by vertical growth (Table 2). None of these changes reached statistical significance. Average serum ALT values were reduced substantially in the follow-up time periods (−45 to −30 U/L), although there was a considerable variability in subject response (standard deviations 65 to 75 U/L) (Table 2). Despite the large interindividual variance, the change in serum ALT was statistically significant in the 1–4 months (*P* = 0.016) and beyond one year (*P* = 0.014) time periods (Table 2). 

**Figure 3:**
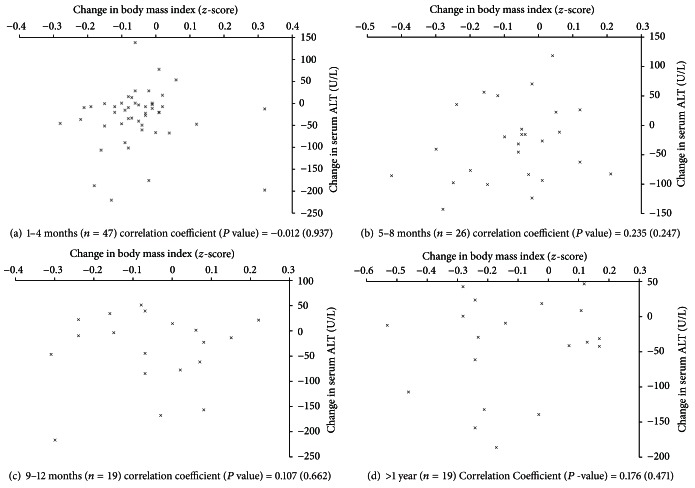
Scatterplot of subject's change in BMI *z*-score and change in serum alanine aminotransferase at time intervals of 1–4 months, 5–8 months, 9–12 months, and greater than 1 year. ALT = alanine aminotransferase. BMI adjusted for age and sex using the 2000 Centers for Disease Control and Prevention growth charts and lambda, mu, and sigma method [17]. The association between changes in BMI *z*-score and changes in serum alanine aminotransferase was assessed by Pearson correlation and is presented as correlation coefficient (*P* value).

The associations between changes in body weight and BMI *z*-score, and serum ALT at each time interval are depicted in Figures 2 and 3. As expected, decreases in body weight and BMI *z*-score generally corresponded to decreases in serum ALT (Table 2). However, the correlations coefficients were small for both the change in body weight (0.076 to 0.371) and BMI *z*-score (−0.012 to 0.235) and did not reach statistical significance at any time interval (Table 2). Adjusting for baseline values in linear regression analysis did not change these findings (Table 2).

## 4. Discussion

 The results of this study provide a preliminary description of pediatric NAFLD in Hawai'i. Asians made up the majority of subjects in this sample, but Whites, Hispanics, and Pacific Islanders were also fairly prevalent, reflecting the diverse ethnic makeup of Hawai'i [23]. Similar to what has been observed elsewhere, subjects were mostly obese, male, and in their second decade of life, and many suffered from other comorbidities, particularly dyslipidemia [2, 4, 5, 13, 25, 26]. Liver biopsy performed in these pediatric patients often revealed inflammation and fibrosis characteristic of steatohepatitis including three subjects with bridging fibrosis [1]. 

 The reason that pediatric NAFLD is more common in males and increases early in the second decade of life is not known, although several mechanisms have been proposed. This period of childhood development is associated with changes in body fat deposition, insulin sensitivity, and hepatic antioxidant defenses, which may confer greater susceptibility to alterations in lipid metabolism and liver disease [27–30]. Boys and girls appear to differ in these physiological changes [27–30].

 These results suggest that pediatric NAFLD patients in Hawai'i generally present with hepatic inflammation and fibrosis and often suffer from other conditions. The amount of hepatic inflammation and fibrosis exceeds that which has been reported in large histological studies conducted in Asian populations and on the mainland United States [1, 31]. However, this was not a representative sample of patients, and the decision to obtain a liver biopsy was biased towards patients that are thought to have more advanced disease. The high prevalence of comorbidities is expected given elevated BMIs in the sample and is consistent with other reports of pediatric NAFLD [4, 5]. Additionally, it is not surprising that dyslipidemias were among the most common comorbidities because NAFLD is a disorder of lipid metabolism [32].

The serum ALT of subjects tended to decrease from the time of referral suggesting improvements in liver disease, although this was not related to changes in body weight or BMI *z*-score. These findings contrast prior clinical trials investigating the effects of weight loss on pediatric NAFLD, which have reported strong associations between reductions in body weight and BMI *z*-score, and indicators of liver disease [14–16]. Unlike the patients in this study, subjects in those clinical trials were volunteers that received comprehensive treatment and monitoring regiments, which are generally not reflective of standard care [14–16]. This distinction is supported by the findings of the trial by Reinehr et al. (2009), which included a control group consisting of children unable to participate in the lifestyle intervention program [16]. While the treatment group had significant reductions in BMI *z*-score, serum ALT, and liver ultrasound at one- and two-year follow-up, the control group remains unchanged for all outcomes [16]. The Treatment of Nonalcoholic Fatty Liver Disease in Children (TONIC) Study, a multicenter clinical trial conducted by the Nonalcoholic Steatohepatitis Clinical Research Network, further supports our findings [33, 34]. Although the TONIC study was designed to investigate the effects of vitamin E and metformin in pediatric NAFLD, all participants received standard diet and exercise advice for weight loss and were monitored over a 96-week period [34]. The placebo group had improvements in serum ALT (−35.2 U/L (95% CI −56.9 to −13.5 U/L)) and histological features of NAFLD despite negligible changes in BMI *z*-score (−0.01 (95% CI −0.08 to 0.06)) and increased body weight (+12.7 kg (95% CI 9.7 to 15.6 kg)) [33]. 

There are several possible explanations for the lack of association between body weight and BMI *z*-score, and serum ALT that was observed in this study. Although adipose tissue, particularly around the viscera, is thought to contribute to the pathogenesis of NAFLD, it is unlikely that the reversal of liver disease associated with weight loss in pediatric NAFLD patients seen in clinical trials can be solely attributed to the shrinking of fat stores [14–16, 35, 36]. Lifestyle interventions targeted towards weight reduction often include recommendations to increase aerobic exercise and reduce dietary intake of added sugars, which may have independent effects on pediatric NAFLD [37, 38]. Furthermore, it is possible that changes in body weight and BMI *z*-score were not sensitive to decreases in body fatness due to increases in muscle mass [39]. Finally, this was a pilot study based on limited sample of patients that had follow-up data, which increases the risk of *β*-errors. This was more pronounced at the later time intervals (contained only 23% of initial sample) and was biased towards subjects that required continued follow-up due to greater disease severity or non-response to treatment (Supplementary Table 1). However, the small correlated coefficients that were observed underscore the weak relationship between these variables.

These findings may reflect an important distinction between efficacy and effectiveness of weight loss in pediatric NAFLD. In the controlled setting of a weight loss intervention trial, many overweight and obese pediatric NAFLD patients were able to achieve clinically relevant reductions in body weight or BMI *z*-score, which were associated with improvements in serological and radiological indicators of liver disease [14–16]. However, in this sample of mostly obese outpatient pediatric NAFLD patients, body weight tended to increase and BMI *z*-score remained similar across the time intervals, a finding that was also observed in the TONIC trial [33]. It is possible that the associations between the changes in body weight and BMI *z*-score, and liver disease only exist in the range of weight loss, that is, generally found in intervention trials. Alternatively, the relationship between the changes in physical activity and diet that reduce liver disease, and changes in body weight and BMI *z*-score may be stronger in the controlled setting of a clinical trial. 

The results of this study indicate that changes in body weight are not an appropriate primary outcome in obese pediatric NAFLD patients. Although body weight is easily measured and may reflect lifestyle changes in experimental conditions, its role in free-living patients treated in the outpatient setting has not been established. Overemphasis on weight reduction, which is unlikely to be maintained in a growing child, may have lasting, negative effects on these patients that have already been identified as being more sensitive to weight-related poor self-esteem [10, 13]. These findings need to be replicated in a larger, prospective study that directly measures diet and physical activity. Based on these findings, greater emphasis should be placed on patient adherence with modifiable behaviors that are thought to contribute to NAFLD.

## Supplementary Material

The relationship between changes in body weight and body mass index (BMI), and serum alanine aminotransferase (ALT) concentrations were assessed in subjects with these measurements taken at time intervals of 1-4 months (58%), 5-8 months (32%), 9-12 months (23%), and beyond one year after the initial visit with the pediatric gastroenterologist (23%). Supplementary Table 1 presents a comparison of subjects who were included in these analyses (Figures 2 and 3) with those subjects who did not have follow-up data. As expected, subjects who were monitored over time tended to have higher baseline serum ALT concentrations. Body weight and BMI status did not appear to a major determinant of patient follow up, although subjects with data available in the 5-8 month time interval had significantly greater baseline body weight (p=0.024).

## Figures and Tables

**Table 1:** Demographic, anthropometric, biochemical, and clinical characteristics of subjects.

Parameter	*n*	Value	Normal range	Abnormal
Age (years)	81	14.1 (11.2–16.2)	N/A	
Sex (*n* (%) Male)	81	70 (86%)	N/A	
Ethnicity (*n* (%))	81		N/A	
American Indian		1 (1%)		
Asian		51 (63%)		
Hispanic		10 (12%)		
Pacific Islander		6 (7%)		
White		13 (16%)		
Body Weight (kg)	81	86.5 ± 26.3	N/A	
Height (m)	81	1.60 ± 0.14	N/A	
BMI (*z*-score)	81	2.30 ± 0.40	<1.64	80/81 (99%)
Glucose, Fasting (mmol/L)	33	5.4 (4.9–5.8)	<6.1	6/33 (18%)
Cholesterol (mmol/L)	34	4.8 (4.1–5.7)	<4.4	21/34 (62%)
LDL-cholesterol (mmol/L)	30	2.7 (2.4–3.4)	<3.4	8/30 (27%)
HDL-cholesterol (mmol/L)	27	1.1 ± 0.3	M > 1.0; F > 1.3	14/27 (52%)
Triglycerides (mmol/L)	32	1.9 (1.5–2.5)	<1.7	20/32 (63%)
Systolic Blood Pressure (*z*-score)	59	1.19 (0.72–1.84)	<1.64	22/59 (37%)
Diastolic Blood Pressure (*z*-score)	59	0.70 ± 1.07	<1.64	
Alanine Aminotransferase (U/L)	81	105 (78–153)	N/A	

BMI adjusted for age and sex using the 2000 Centers for Disease Control and Prevention growth charts and lambda, mu and sigma method [17].

Systolic and diastolic blood pressures adjusted for age, sex, and height as per the National High Blood Pressure Education Program Working Group on High Blood Pressure in Children and Adolescents recommendations [18].

Values are presented as number (percent) for categorical variables, mean ± standard deviation for parametric continuous variables and median (interquartile range) for nonparametric continuous variables. Continuous variables were classified as nonparametric based on Shapiro-Wilk test and confirmed by analysis of frequency distribution graphs.

Normal ranges as per Schwimmer et al. (2003) and Graham et al. (2009) [19, 20]. Abnormal values are frequency (percent) of subjects outside the normal range. Hypertension was present if either systolic or diastolic values were above normal [20].

**Table 2:** Effectiveness of weight loss treatment in children with nonalcoholic fatty liver disease.

Outcome	Δ Weight (kg)	Δ Height (m)	Δ BMI (*z*-score)	Δ ALT (U/L)
*T*_1–4_ (*n* = 47)				
Value	−0.2 ± 2.9 (0.976)	+0.01 ± 0.01 (0.740)	−0.05 ± 0.11 (0.532)	−32 ± 66 (0.016)
Correlation	0.076 (0.611)		−0.012 (0.937)	
Regression	4.72 (0.107)		68.8 (0.377)	
*T*_5–8_ (*n* = 26)				
Value	+1.4 ± 4.8 (0.825)	+0.02 ± 0.02 (0.583)	−0.07 ± 0.15 (0.493)	−30 ± 65 (0.134)
Correlation	0.360 (0.071)		0.235 (0.247)	
Regression	3.28 (0.187)		101.1 (0.213)	
*T*_9–12_ (*n* = 19)				
Value	+3.3 ± 4.7 (0.680)	+0.03 ± 0.03 (0.425)	−0.05 ± 0.15 (0.718)	−37 ± 75 (0.157)
Correlation	0.077 (0.753)		0.107 (0.662)	
Regression	1.08 (0.617)		51.3 (0.424)	
*T*_>1_ (*n* = 19)				
Value	+7.1 ± 9.0 (0.471)	+0.07 ± 0.06 (0.219)	−0.12 ± 0.21 (0.453)	−45 ± 69 (0.014)
Correlation	0.371 (0.118)		0.176 (0.471)	
Regression	3.10 (0.073)		69.0 (0.320)	

*T*_1–4_: time interval 1–4 months, *T*_5–8_: time interval 5–8 months, *T*_9–12_: time interval 9–12 months, *T*_>1_: time interval beyond one year, SD: standard deviation, ALT: alanine aminotransferase.

BMI adjusted for age and sex using the 2000 Centers for Disease Control and Prevention growth charts and lambda, mu and sigma method [17].

Values are the change in parameter presented as mean ± standard deviation with *P* value in parentheses based on independent two-sample *t*-test and Wilcoxon rank sum test for parametric and nonparametric continuous variables, respectively.

Association between changes in body weight and BMI *z*-score, and changes in serum alanine aminotransferase assessed by Pearson correlation and linear regression and presented as correlation coefficients and regression coefficients with *P* values in parentheses. Linear regression adjusted for initial serum alanine aminotransferase, and baseline values for body weight and BMI *z*-score of the corresponding analysis.
