# Supplementary material for: The Effect of Weight Loss on Pediatric Nonalcoholic Fatty Liver Disease
Source: ISRN Gastroenterol. 2013 May 27;2013:398297. doi: 10.1155/2013/398297 (PMC3678446; doi:10.1155/2013/398297)
Supplement: Supplementary file 1 — The relationship between changes in body weight and body mass index (BMI), and serum alanine aminotransferase (ALT) concentrations were assessed in subjects with these measurements taken at time intervals of 1-4 months (58%), 5-8 months (32%), 9-12 months (23%), and beyond one year after the initial visit with the pediatric gastroenterologist (23%). Supplementary Table 1 presents a comparison of subjects who were included in these analyses (Figures 2 and 3) with those subjects who did not have follow-up data. As expected, subjects who were monitored over time tended to have higher baseline serum ALT concentrations. Body weight and BMI status did not appear to a major determinant of patient follow up, although subjects with data available in the 5-8 month time interval had significantly greater baseline body weight (p=0.024). [file 398297.f1.pdf]

Supplementary Table 1. Comparison of Subjects Included and Not Included in the Analysis According to Time Interval

**Parameter**	**Included**	**Not Included**	**p-value**
1-4 Months			
Sample Size	47	34	
Body Weight (kg)	86.5 ± 25.4	86.4 ± 27.8	0.984
BMI (z-score)	2.29 ± 0.37	2.31 ± 0.45	0.817
Alanine Aminotransferase (U/L)	124 (79-189)	92 (73-122)	0.026
5-8 Months			
Sample Size	26	55	
Body Weight (kg)	76.9 ± 23.2	91.0 ± 26.6	0.024
BMI (z-score)	2.18 ± 0.40	2.35 ± 0.40	0.072
Alanine Aminotransferase (U/L)	124 (96-196)	100 (73-148)	0.026
9-12 Months			
Sample Size	19	62	
Body Weight (kg)	82.0 ± 25.4	87.8 ± 26.6	0.379
BMI (z-score)	2.17 ± 0.45	2.33 ± 0.38	0.120
Alanine Aminotransferase (U/L)	109 (77-196)	104 (78-152)	0.359
Beyond 1 Year			
Sample Size	19	62	
Body Weight (kg)	79.4 ± 31.3	88.6 ± 24.4	0.185
BMI (z-score)	2.37 ± 0.48	2.27 ± 0.38	0.240
Alanine Aminotransferase (U/L)	118 (88-198)	102 (77-151)	0.056

Legend – Supplementary Table 1

BMI adjusted for age and sex using the 2000 Centers for Disease Control and Prevention growth charts and lambda, mu and sigma method.[18]
Values are presented as mean ± standard deviation for parametric continuous variables and median (interquartile range) for nonparametric continuous variables. Continuous variables were classified as nonparametric based on Shapiro-Wilk test, and confirmed by analysis of frequency distribution graphs. Comparison of groups was conducted using independent two-sample t-tests and Wilcoxon rank sum tests for parametric and non-parametric variables, respectively.
